# Transmission of Leprosy by Flies

**Published:** 1916-10

**Authors:** 


**Transmission of Leprosy by Flies.** E. Marchoux, Annales de l'Inst. Pasteur, Feb., states that flies (Diptera) carry the germs on the feet and proboscis, that the germs do not die in the flies' intestine, that a fly does not defaecate every time it feeds, that the danger of infection is local, not because flies do not travel to a considerable distance but because on long flights, the germs are detached and die.

